# Toxic Effects of Vanillic Acid and Sinapic Acid on *Spodoptera frugiperda*

**DOI:** 10.3390/biology14080979

**Published:** 2025-08-01

**Authors:** Ya-Nan Deng, Jin-Yan Lv, Xiao-Rong Liu, Dan Niu, Ling-Xin Xu, Jun-Xin Yan

**Affiliations:** 1 Dazhou Key Laboratory of Agricultural Resources Development and Ecological Conservation in Daba Mountain, Sichuan University of Arts and Science, Dazhou 635000, China; 2 College of Landscape Architecture, Northeast Forestry University, Harbin 150040, China

**Keywords:** plant-derived secondary compounds, fall armyworm, ecofriendly control, growth, development

## Abstract

The tolerance of the fall armyworm (*Spodoptera frugiperda*) to plant-derived secondary compounds gradually increases with instars. Therefore, even if plant-based additives are applied at early stages, such as the second or third instar, they may have a differential impact on the ecofriendly control of *S. frugiperda*. In this study, *S. frugiperda* larvae were exposed to vanillic acid or sinapic acid at the second and third instar, and physiological and growth parameters were measured. The results showed that the effects of vanillic acid treatment on *S. frugiperda* were similar at the different instars. They can significantly affect the larval carboxylesterase, glutathione S-transferase, and mixed-function oxidase activities. By reducing larval food intake, food conversion, and utilization efficiency while increasing the food consumption rate, it inhibits weight accumulation. This leads to a significant extension of the development of both the larval and pupal stages, and the adult longevity was reduced. Treatment with sinapic acid at the second instar extended the negative effects on the pupal duration of *S. frugiperda* when compared to treatment at the third instar, but did not affect adult longevity. Therefore, vanillic acid treatment at the second or third instar stage, can play an important role in the ecofriendly control process of *S. frugiperda*. The results of this study are of great significance for integrated pest management.

## Simple Summary

Herbivorous insects may gradually increase their tolerance to plant-derived compounds. Therefore, the timing of their application may influence their efficacy in pest control. This study found that at the 2nd or 3rd instar stage of a fall armyworm, exposure to vanillic acid had a similar effect on its 4th–6th instar larvae, pupae, and adult stages. Both treatments significantly affected the detoxification enzyme activity of the larvae. By reducing food intake, utilization, and larval weight, it can further prolong the development of the larvae and pupae, as well as the longevity of the adult. However, due to the strong degradation and isolation of sinapic acid by the detoxifying enzyme of the fall armyworm, the larvae developed a strong tolerance. Exposure to sinapic acid, even at the 2nd instar, only significantly prolonged its pupal duration and had no adverse effects on its adult longevity.

## 1. Introduction

According to statistics, the annual crop yield reduction caused by pest feeding is close to 20% of the total global agricultural production [1]. Accordingly, pest stress has become one of the main factors affecting the rapid development of the global agricultural economy [1,2,3]. Specifically, the spread of super pests such as the fall armyworm (*Spodoptera frugiperda*) has exacerbated the limiting effect of pest stress on the agricultural economy [4,5,6,7]. This has led to panic among agricultural growers, and they often overuse pesticides in order to achieve better pest control during field production [8,9,10]. The frequent and unrestrained use of pesticides not only increases the cost of agricultural cultivation but also challenges the effectiveness of these pesticides because of a growing resistance in the pests [7]. This is most significant in the control of lepidopteran pests [4,5,11]. In addition to these direct issues, the chemical control of pests also brings some indirect problems, such as the discovery that neonicotinoid insecticides have toxic effects on bees [12]. There is also evidence to suggest that nearly three-quarters of pesticides can be transferred through drift, surface runoff, and soil leaching after spraying, thereby polluting the environment or endangering nearby bodies of water [13,14]. What is most worrisome is the absorption and accumulation of pesticides by plants, which may lead to the migration of residual chemicals into the food chain. This greatly increases the risk of food safety, raises the incidence rate of cancer and the probability of neonatal malformations, seriously threatening public health [10]. The economic and environmental problems caused by pest invasion are driving an increasing demand for ecofriendly pest control strategies.

Some recent studies have found that some plant secondary metabolites, such as tannin, rutin, chlorogenic acid, naringin, and other compounds, can interfere with the feeding and growth of herbivorous pests through toxic effects, achieving effective prevention and control [15,16,17,18]. Tannin can increase the levels of malondialdehyde and hydrogen peroxide in the body of the fall webworm (*Hyphantria cunea*), while also inhibiting their antioxidant and detoxifying enzyme activities [19]. This causes serious oxidative damage to their larvae, inhibits their utilization and growth of food, and reduces their harmfulness. Moreover, these plant-derived secondary compounds decompose quickly in the environment and leave less residue when compared to traditional pesticides, making them suitable for regular use as pesticide enhancers or substitutes [19]. This would thereby reduce the use of highly toxic chemical pesticides in the integrated pest management processes, making them potentially useful in an ecofriendly pest control solution [20,21,22].

Given the increased feeding and detoxification capabilities of *S. frugiperda* in its later instars, early-stage applications may yield better control efficacy [23,24,25]. Most newly hatched larvae of these pests feed on eggshells and rapidly grow by feeding on host plants after the second instar [4,7]. When they reach the third instar, whether or not the changes in tolerance will have a direct impact on the effectiveness of plant-based additives remains to be studied and verified.

In this study, *S. frugiperda*, an invasive phytophagous pest, was selected as the research object. Based on preliminary experimental results, vanillic acid and sinapic acid, which can interfere with the metabolism of *S. frugiperda*, were selected as plant-derived additives [26,27]. The two phenolic compounds were added to the artificial diet of the 2nd and 3rd instar stages of *S. frugiperda*, and the detoxifying enzyme activity, food intake, food utilization, weight changes, as well as developmental duration of the larvae (4th–6th instars) and pupae, and adult longevity were measured after feeding. Principal component analysis (PCA) was used to reveal the internal structure among these variables, and a new comprehensive index was formed to evaluate the effects of phenolic compounds treatment at different instars on the growth and physiology of *S. frugiperda*, providing further guidance for their control.

## 2. Materials and Methods

### 2.1. Rearing and Treatment of Insects

*S. frugiperda* eggs and artificial diet were obtained from Henan Jiyuan Baiyun Industry Co., Ltd. (Jiyuan, China). The eggs were hatched in an environment with a temperature of 25 °C ± 1 °C at a relative humidity of around 40–50%, photoperiod of 16L:8D, and light intensity of 12,000 lux. The larvae were reared under the same environmental conditions after hatching. Each *S. frugiperda* egg mass can hatch approximately 150 larvae. These larvae were reared in a plastic box (top diameter 120 mm, bottom diameter 82 mm, height 60 mm). To prepare the feed, 700 mL of distilled water was heated to boiling, and then 300 g of dry artificial diet powder was immediately added. After cooking, the artificial diet was cooled to room temperature. Vanillic acid (Macklin, Shanghai, China. CAS: 121-34-6) or sinapic acid (Source Leaf Biotechnology, Shanghai, China. CAS: 530-59-6) was added to the unsolidified feed of treatment groups at an effective dose of 15 mg/g, respectively [28]. The experiment consisted of a total of 6 groups. A total of 800 larvae were reared in each group. The control group (CK_1_, CK_2_) fed only on the artificial diet without plant-based additives during the larval stage, while the rest were the treatment groups. The newly hatched larvae in the treatment group were the same as those in the control group and were fed with a plant-based additive free artificial diet. However, after the larvae molted to the 2nd instar, the feed was switched to that supplemented with vanillic acid or sinapic acid until the larvae pupated, and were recorded as the V_2_ and S_2_ groups, respectively. The V_3_ and S_3_ treatment groups, alternatively, switched to the supplemented feed containing vanillic acid or sinapic acid after the larvae molted to the 3rd instar. The treatment and sampling stages are presented in Table 1. The plastic boxes used for rearing the larvae were replaced every two days, and the artificial diet was changed daily. When each group of larvae molted to the 3rd instar, they were all moved to be reared separately (top diameter 73, bottom diameter 54, height 40 mm).

### 2.2. Determination of Detoxification Enzyme Activity of S. frugiperda Larvae

Larvae from the 4th, 5th, or 6th instar that had molted within the past 48 h were selected from both the treatment and control groups and then preserved by quick freezing at −80 °C in order to determine the detoxifying enzyme activity. The experiment was set up with a total of 3 replicates in each group, with 3 larvae per replicate being treated collectively. Using 2 mL of normal saline (0.9%) as the extraction solution, the larvae were ground into a homogenate under an ice bath and centrifuged for 10 min at 10,000 rpm at 4 °C to obtain the enzyme liquid to be measured. The determination of glutathione S-transferase (GST) activity was based on Halmenschelager et al. [29]. The activities of mixed-function oxidase (MFO) and carboxylesterase (CarE) was based on the study of Lazarevic et al. [30]. The activity units of CarE is min/mg of protein, while the active units of GST and MFO are both nmol/min/mg of protein.

### 2.3. Determination of Food Utilization of S. frugiperda Larvae

When each group of larvae developed to the 4th, 5th, and 6th instar, newly molted larvae were selected. The changes in food weight and larval weight within 24 h before and after feeding were recorded. The feces discharge quality was also measured. A total of 3 replicates in each group were set, with 10 larvae per replicate. Subsequently, following the method of Tan et al. [19] and Zhang et al. [24], the changes in the food intake, the food consumption rate, food conversion rate, and food utilization rate of the larvae were calculated, respectively, according to the following formulas.

Food intake (mg) = (food weight before feeding − residual food weight after feeding)/(1 − corrected water loss rate)(1)

Food consumption rate (%) = (food intake − weight of feces)/food intake × 100%(2)

Food conversion rate (%) = (larval weight after feeding − larval weight before feeding)/(food intake − feces discharge quality) × 100%(3)

Food utilization rate (%) = (larval weight after feeding − larval weight before feeding)/food intake × 100%(4)

### 2.4. Determination of Larval Weight and Developmental Duration of S. frugiperda

The 4th, 5th, or 6th instar larvae within 48 h of new molting in each group were weighed. Each group of 10 larvae was weighed, and their average weight was considered as one replicate. A total of 3 replicates were set in each control or treatment group. The developmental duration of the larvae, the pupal duration, or the adult longevity were recorded every 12 h. A total of 3 replicates were set for each group, each of which included 100 *S. frugiperda*. During the experiment, each adult was reared separately in an insect cage (35 cm × 35 cm × 35 cm). Honey water (10% *w*/*v*) was provided as a nutritional source until adult death.

### 2.5. Statistical Analysis

After summarizing the data, SPSS 19.0 software was used for data analysis. Levene’s test was conducted for homogeneity of variance, and Tukey’s HSD method of one-way ANOVA was used for significance analysis of differences between data. Subsequently, a comprehensive analysis of the individual indicators was conducted using principal component analysis (PCA), and finally, images were constructed using Origin 2021.

## 3. Results

### 3.1. Detoxification Enzyme Activity of S. frugiperda Exposure to Phenols

**Figure 1:**
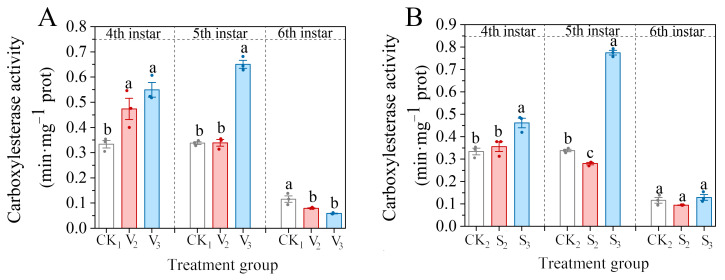
Changes in the carboxylesterase (CarE) activity of the 4th–6th instar larvae of *S. frugiperda* after exposure to vanillic acid (**A**) or sinapic acid (**B**) at the 2nd or 3rd instar. Values in the figure are mean ± standard error. According to Tukey’s HSD test, there is no significant difference between the same letters (*p* > 0.05).

The CarE activity of the fourth instar larvae significantly decreased following treatment with vanillic acid (V_2_, V_3_) (*F* = 12.695, df = 2, *p* = 0.007), reaching 141.92% and 164.57% of the control group, respectively (Figure 1A). After the larvae reached the fifth instar, the enzyme activity in the V_2_ group neared the same level as the control, while the enzyme activity in the sixth instar larvae significantly decreased (*F* = 14.741, df = 2, *p* = 0.005), reaching 68.75% of the control. However, in the V_3_ treatment group, the enzyme activity of the fifth instar larvae significantly increased. The enzyme activity of the sixth instar larvae significantly decreased, reaching 50.76% of the control.

The changes in the CarE activity in the fourth instar larvae of sinapic acid treatment groups (S_2_, S_3_) were similar to those of vanillic acid, and the S_3_ treatment group was significantly higher than the control level, 138.26% (*F* = 12.113, df = 2, *p* = 0.008), but the enzyme activity in the fifth and sixth instar larvae of the S_2_ group was inhibited (Figure 1B).

**Figure 2:**
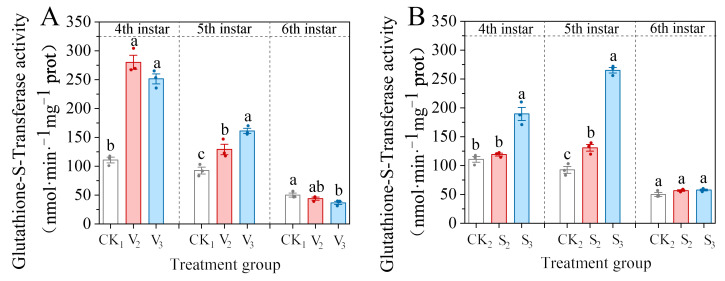
Changes in the glutathione-S-transferase (GST) activity of the 4th–6th instar larvae of *S. frugiperda* after exposure to vanillic acid (**A**) or sinapic acid (**B**) at the 2nd or 3rd instar. Values in the figure are mean ± standard error. According to Tukey’s HSD test, there is no significant difference between the same letters (*p* > 0.05).

In the vanillic acid (V_2_, V_3_) treatment group, the GST activity in the fourth and fifth instar larvae significantly increased (4th: *F* = 99.643, df = 2, *p* < 0.001; 5th: *F* = 26.279, df = 2, *p* = 0.001) (Figure 2A). However, after the larvae developed to the sixth instar, enzyme activity decreased, and V_3_ was significantly lower than the control (*F* = 5.299, df = 2, *p* = 0.047), accounting for 73.18% of the control.

The S_3_ treatment with sinapic acid significantly increased the GST activity of the fourth and fifth instar larvae (4th: *F* = 34.463, df = 2, *p* = 0.001; 5th: *F* = 268.796, df = 2, *p* < 0.001), which were 170.58% and 286.01% of the control, respectively. However, the S_2_ group only showed a significant increase in enzyme activity in the fifth instar larvae, which was 141.28% of the control (Figure 2B). The trend of enzyme activity changes in the sixth instar larvae in the S_2_ and S_3_ treatment groups was consistent, similar to the control (*F* = 3.308, df = 2, *p* = 0.108).

**Figure 3:**
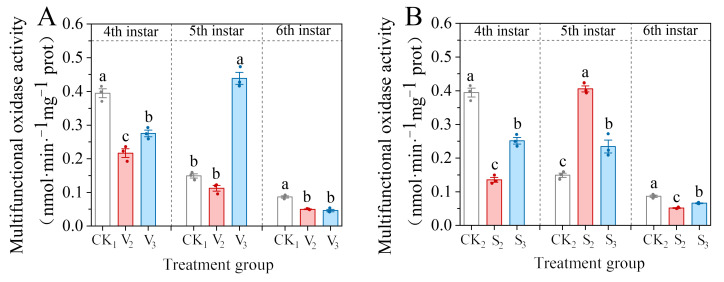
Changes in the mixed-function oxidase (MFO) activity of the 4th–6th instar of *S. frugiperda* after exposure to vanillic acid (**A**) or sinapic acid (**B**) at the 2nd or 3rd instar. Values in the figure are mean ± standard error. According to Tukey’s HSD test, there is no significant difference between the same letters (*p* > 0.05).

The vanillic acid treatment (V_2_, V_3_) significantly inhibited the MFO activity of the fourth instar larvae of *S. frugiperda* (*F* = 57.544, df = 2, *p* < 0.001), and the enzyme activity of the fifth instar larvae still showed a decreasing trend under V_2_ treatment, but did not reach a significant difference compared with the control (Figure 3A). The enzyme activity of the fifth instar larvae in the V_3_ treatment group showed a significant increase (*F* = 224.981, df = 2, *p* < 0.001) of 294.46% of the control. However, both treatments significantly inhibited the enzyme activity of the sixth instar larvae (*F* = 57.917, df = 2, *p* < 0.001), decreasing to 57.60% and 53.27% of the control, respectively.

The effect of the sinapic acid treatment (S_2_, S_3_) on the MFO activity in the fourth and sixth instar larvae was similar to that of the vanillic acid treatment (Figure 3B). However, the enzyme activity of the fifth instar larvae in the sinapic acid treatment group significantly increased (*F* = 105.078, df = 2, *p* < 0.001), with the enzyme activity of larvae treated with S_2_ reaching 272.43% of the control, while S_3_ treatment was 157.52% of the control.

### 3.2. Food Utilization of S. frugiperda Larvae After Exposure to Phenols

**Figure 4:**
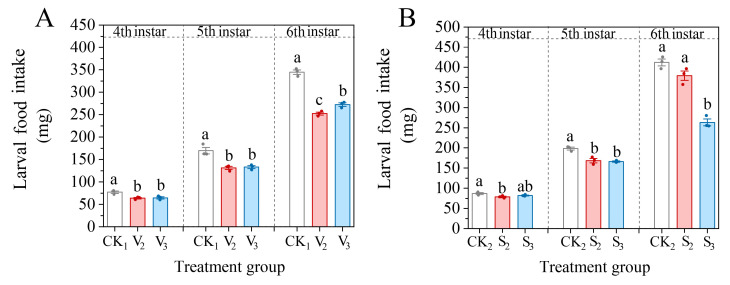
Changes in the food intake of the 4th–6th instar larvae of *S. frugiperda* after exposure to vanillic acid (**A**) or sinapic acid (**B**) at the 2nd or 3rd instar. Values in the figure are mean ± standard error. According to Tukey’s HSD test, there is no significant difference between the same letters (*p* > 0.05).

After vanillic acid or sinapic acid treatment to the second or third instar stage of *S. frugiperda*, the food intake of the 4th–6th instars larvae was inhibited (Figure 4). Compared to the control group, the S_2_ treatment had a significant inhibitory effect on the food intake of only the fourth and fifth instars, which were 90.45% and 84.84% of the control, respectively (4th: *F* = 5.231, df = 2, *p* = 0.048; 5th: *F* = 23.157, df = 2, *p* = 0.002).

**Figure 5:**
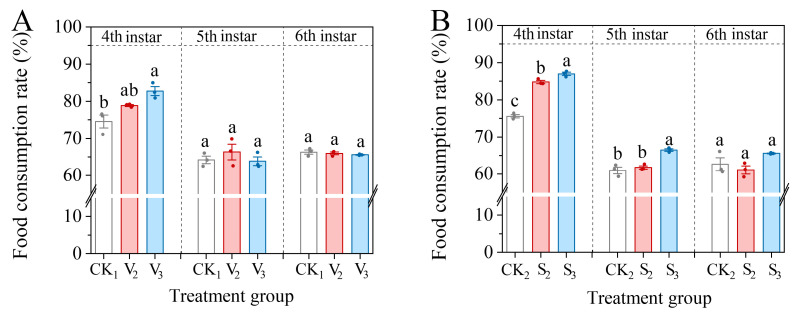
Changes in the food consumption rate of the 4th–6th instar larvae of *S. frugiperda* after exposure to vanillic acid (**A**) or sinapic acid (**B**) at the 2nd or 3rd instar. Values in the figure are mean ± standard error. According to Tukey’s HSD test, there is no significant difference between the same letters (*p* > 0.05).

Compared with the control group, the food consumption rate of the fourth instar larvae in the vanillic acid treatment (V_2_, V_3_) and sinapic acid treatment (S_2_, S_3_) groups showed an increase (Figure 5). However, only the V_3_ group was significantly higher than the control in the vanillic acid treatment (*F* = 10.987, df = 2, *p* = 0.01), while the consumption rate of the fourth instar larvae in the S_2_ and S_3_ treatments significantly increased (*F* = 192.454, df = 2, *p* < 0.001). When the larvae developed to the fifth or sixth instar, although the consumption rates of the V_2_ and V_3_ treatments either slightly increased or decreased when compared to the control, the difference was not significant (5th: *F* = 0.791, df = 2, *p* = 0.496; 6th: *F* = 0.825, df = 2, *p* = 0.482). The consumption rate of the fifth instar larvae in S_3_ treatment still showed a significant increase (*F* = 24.915, df = 2, *p* = 0.001), but after the larvae developed to sixth instar, the consumption rate in S_2_ and S_3_ treatment groups tended towards the rate of the control group (*F* = 2.723, df = 2, *p* = 0.144).

**Figure 6:**
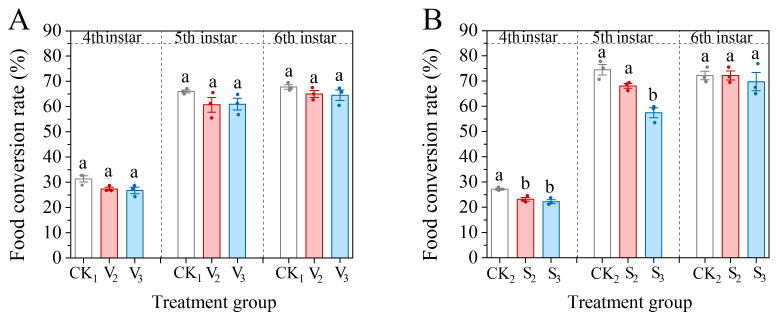
Changes in the food conversion rate of the 4th–6th instar larvae of *S. frugiperda* after exposure to vanillic acid (**A**) or sinapic acid (**B**) at the 2nd or 3rd instar. Values in the figure are mean ± standard error. According to Tukey’s HSD test, there is no significant difference between the same letters (*p* > 0.05).

The conversion rates of the 4th–6th instar larvae in the vanillic acid treatment groups (V_2_, V_3_) were all reduced, but did not reach a significant difference (4th: *F* = 5.057, df = 2, *p* = 0.052; 5th: *F* = 1.881, df = 2, *p* = 0.232; 6th: *F* = 1.344, df = 2, *p* = 0.329) (Figure 6). The food conversion rate of fourth instar larvae in the sinapic acid treatment (S_2_, S_3_) groups significantly decreased (*F* = 15.903, df = 2, *p* = 0.004), reaching 23.14% and 22.19%, respectively. After reaching the fifth instar, only the S_3_ treatment group showed a significant decrease in food conversion rate (*F* = 23.818, df = 2, *p* = 0.001), reaching 57.44%. However, the conversion rate of sixth instar larvae tended towards the level of the control (*F* = 0.313, df = 2, *p* = 0.743).

**Figure 7:**
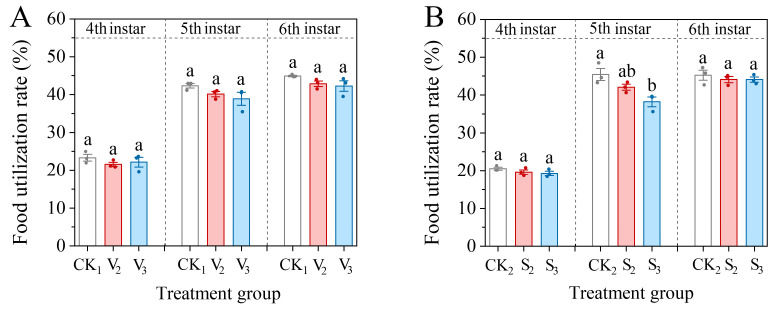
Changes in the food utilization rate of the 4th–6th instar larvae of *S. frugiperda* after exposure to vanillic acid (**A**) or sinapic acid (**B**) at the 2nd or 3rd instar. Values in the figure are mean ± standard error. According to Tukey’s HSD test, there is no significant difference between the same letters (*p* > 0.05).

The food utilization rate of the 4th–6th instar larvae decreased following treatment with vanillic acid (V_2_, V_3_), but the difference was not significant (4th: *F* = 0.897, df = 2, *p* = 0.456; 5th: *F* = 2.361, df = 2, *p* = 0.175; 6th: *F* = 2.258, df = 2, *p* = 0.186) (Figure 7A). The sinapic acid treatment (S_2_, S_3_) also had an inhibitory effect on the food utilization efficiency of larvae. Only the food utilization efficiency of the fifth instar larvae treated with S_3_ showed any significant difference, with a decrease of 38.20% (*F* = 8.139, df = 2, *p* = 0.02) (Figure 7B).

### 3.3. Larval Body Weight of S. frugiperda After Exposure to Phenols

**Figure 8:**
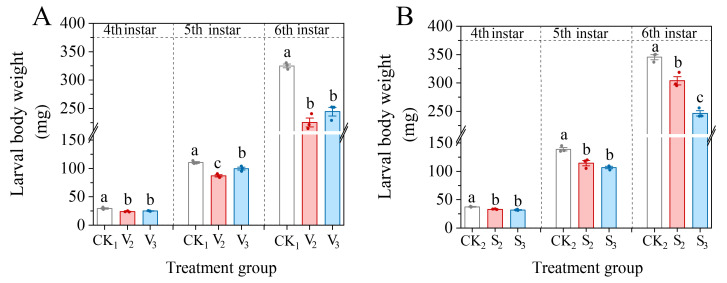
Changes in larval body weight of the 4th–6th instar larvae of *S. frugiperda* after exposure to vanillic acid (**A**) or sinapic acid (**B**) at the 2nd or 3rd instar. Values in the figure are mean ± standard error. According to Tukey’s HSD test, there is no significant difference between the same letters (*p* > 0.05).

After the 2nd or 3rd instar stage of *S. frugiperda* exposed to vanillic acid or sinapic acid, the body weight of the 4th–6th instar larvae was significantly reduced (Figure 8). In particular, the sixth instar larvae were 69.35% and 75.17% of the control in V_2_ and V_3_ treatment (*F* = 63.079, df = 2, *p* < 0.001), and 87.96% and 71.28% of the control in S_2_ and S_3_ treatment (*F* = 76.980, df = 2, *p* < 0.001), respectively.

### 3.4. Developmental Duration of S. frugiperda Larvae After Exposure to Phenols

**Figure 9:**
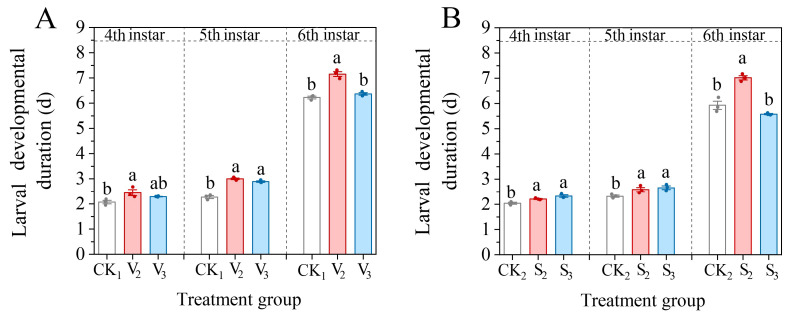
Changes in the developmental duration of the 4th–6th instar larvae of *S. frugiperda* after exposure to vanillic acid (**A**) or sinapic acid (**B**) at the 2nd or 3rd instar. Values in the figure are mean ± standard error. According to Tukey’s HSD test, there is no significant difference between the same letters (*p* > 0.05).

The vanillic acid treatment (V_2_, V_3_) prolonged the developmental duration of the 4th–6th instar larvae of *S. frugiperda* (4th: *F* = 5.871, df = 2, *p* = 0.039; 5th: *F* = 84.683, df = 2, *p* < 0.001; 6th: *F* = 50.807, df = 2, *p* < 0.001), but the V_2_ treatment group was significantly higher than the control, while the V_3_ treatment only significantly increased the developmental duration of the fifth instar larvae (Figure 9A). Compared with the control, the sinapic acid treatment (S_2_, S_3_) significantly increased the developmental duration of the fourth and fifth instar larvae (4th: *F* = 13.892, df = 2, *p* = 0.006; 5th: *F* = 6.458, df = 2, *p* = 0.032), with the S_2_ treatment also significantly prolonging the developmental duration of the sixth instar larvae (Figure 9B).

### 3.5. Pupal Duration of S. frugiperda After Exposure to Phenols

**Figure 10:**
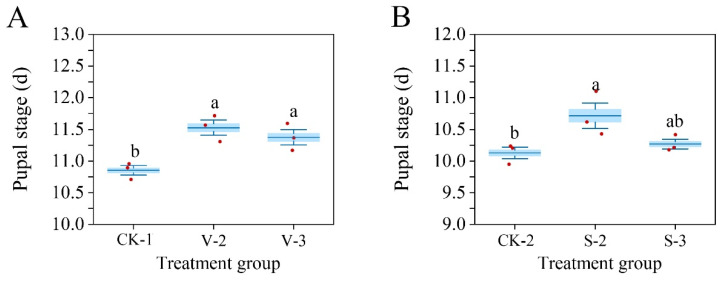
Changes in the pupal duration of *S. frugiperda* after exposure to vanillic acid (**A**) or sinapic acid (**B**) at the 2nd or 3rd instar. Values in the figure are mean ± standard error. According to Tukey’s HSD test, there is no significant difference between the same letters (*p* > 0.05).

After vanillic acid or sinapic acid treatment at the second or third instar stage of *S. frugiperda*, the pupal duration was prolonged (Figure 10). Compared with the control group, both vanillic acid V_2_ and V_3_ treatments significantly prolonged the pupal duration (*F* = 10.833, df = 2, *p* = 0.01), while sinapic acid treatment only significantly prolonged the S_2_ pupal duration (*F* = 5.207, df = 2, *p* = 0.049).

### 3.6. Adult Longevity of S. frugiperda After Exposure to Phenols

**Figure 11:**
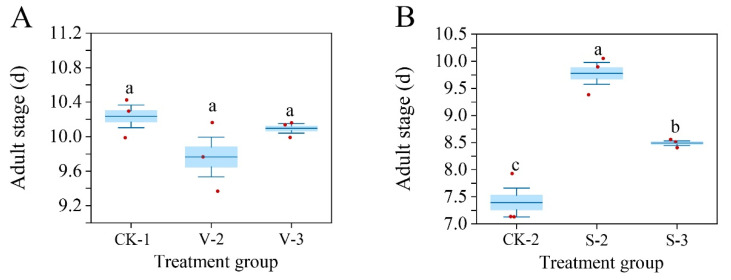
Changes in the adult longevity of *S. frugiperda* after exposure to vanillic acid (**A**) or sinapic acid (**B**) at the 2nd or 3rd instar. Values in the figure are mean ± standard error. According to Tukey’s HSD test, there is no significant difference between the same letters (*p* > 0.05).

After the second or third instar larvae of *S. frugiperda* were exposed to vanillin acid, the adult longevity was slightly shorter than the control but the difference was not significant (*F* = 2.425, df = 2, *p* = 0.169) (Figure 11A). However, after exposure to sinapic acid to the second or third instar stage of *S. frugiperda*, the adult longevity was significantly prolonged compared to the control group (*F* = 37.491, df = 2, *p* < 0.001) (Figure 11B).

### 3.7. Comprehensive Analysis of the Effects of S. frugiperda After Exposure to Phenols

**Figure 12:**
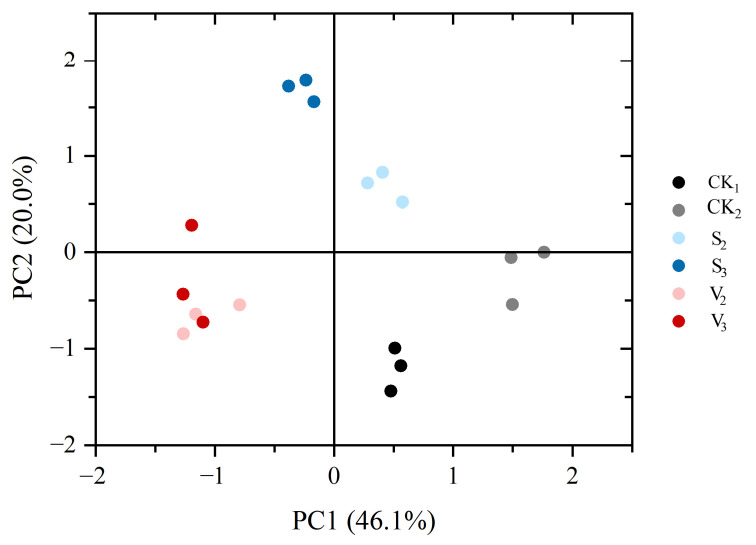
Principal component analysis (PCA) plot, showing the differences between the control group and the treatment group.

According to the results of principal component analysis, the principal components (PC1 and PC2) explained approximately 70% (20% and 46.1%) of the variation (Figure 12). The food intake (4th instar: 0.928, 5th instar: 0.894, 6th instar: 0.732) and body weight (4th instar: 0.954, 5th instar: 0.846, 6th instar: 0.706) of the larvae contributed significantly to PC1, while MFO activity (4th instar: 0.661, 6th instar: 0.572), food conversion rate (4th instar: 0.863, 5th instar: 0.644), and utilization rate (4th instar: 0.631, 5th instar: 0.668) contributed significantly to PC2. The data of the vanillic acid (V_2_, V_3_) and sinapic acid (S_2_, S_3_) treatment groups were well separated from the control group. However, the degree of separation between the vanillic acid (V_2_, V_3_) treatment group and the control group was mainly higher in PC1, while the sinapic acid (S_2_, S_3_) treatment group and the control group were mainly higher in PC2. The degree of separation between V_2_ and V_3_ was relatively low, while the degree of separation between S_2_ and S_3_ was relatively high.

## 4. Discussion

Plant-derived secondary compounds can often serve as ecofriendly potential control substances of herbivorous pests [23]. But the timing of application may influence their efficacy in pest control. Therefore, in this study, the second and third instar of *S. frugiperda* larvae were exposed to vanillic acid or sinapic acid. By analyzing the changes in physiological and growth parameters of the *S. frugiperda* after feeding, the potential impact of exposure to the phenolic compounds at different instars was evaluated.

One of the important defense mechanisms for herbivorous pests to resist damage from plant-derived secondary compounds is to activate detoxification mechanisms [24,25]. These detoxifying enzymes can degrade or isolate toxic substances through oxidation, hydroxylation, and other processes, reducing toxic damage [31,32]. For example, silkworm (*Bombyx mori*) larvae resist the toxic effects of quercetin by increasing the activity of defense enzymes such as CarE and GST [33]. This self-protection method of herbivorous pests may increase the difficulty of integrated pest management. Additionally, in the study on toxic compounds targeting the common cutworm (*S. litura*), it was found that phenolic compounds such as ferulic acid, quinic acid, caffeic acid, and kaempferol had similar effects, all of which inhibited the detoxification enzyme activity of *S. litura* larvae [15]. The results of the current study are similar, but not entirely identical. Whether in the second or third instar stage of *S. frugiperda* larvae, the application of vanillic acid (V_2_, V_3_) significantly inhibited the activities of CarE, GST, and MFO in the sixth instar larvae. The sinapic acid treatment group (S_2_, S_3_) mainly inhibited the MFO activity of fourth and sixth instar larvae, while the activities of the three enzymes in the remaining larval stages showed significant increases. The structural differences between the two phenolic compounds may have an impact on the degradation or isolation rate of detoxifying enzymes in larvae [31,32]. This indicates that there is a difference in the tolerance of *S. frugiperda* to two plant-based additives. Nevertheless, it can be clearly stated that whether applied at the second or third instar stage, vanillic acid and sinapic acid caused toxic effects on *S. frugiperda*, and the larvae mainly alleviated the toxic damage by regulating enzyme detoxification activity.

In addition, some studies have shown that when the dose of plant-based additives is appropriate, it can limit the growth and development of herbivorous pests [15,23]. This is due to the fact that phenolic substances not only interfere with the defense and detoxification mechanisms of feeding pests, but also limit the abundance of gut microbiota related to digestion in their bodies, further interfering with their nutrient absorption and metabolism [19]. In fact, our study results also found that the self-protection strategy of the larvae attempting to alleviate toxic damage by regulating detoxifying enzyme activity is not entirely effective. The development duration of the larvae in the vanillic acid (V_2_, V_3_) and sinapic acid (S_2_, S_3_) treatment groups were significantly prolonged, which was particularly evident after vanillic acid treatment at the second instar of larvae. The food intake of the treated larvae significantly decreased, and the food consumption rate increased, but the food conversion rate and utilization rate declined. This led to a decrease in the weight gain of the larvae and prolonged their developmental period. However, the larval stage is the main stage for the accumulation of energy through feeding for *S. frugiperda*, especially in the sixth instar stage, where their intake can reach the sum of all other instar stages [10]. At this time, insufficient nutrient acquisition not only limits the growth of the larvae, but also directly negatively affects the subsequent developmental process [34,35,36]. In our study, regardless of whether the second or third instar larvae are exposed to vanillic acid, the pupal duration of *S. frugiperda* is significantly prolonged, and the adult longevity is significantly shortened. This may increase the risk of exposure of its larvae and pupae to natural enemies, adverse climate factors, or other control measures, and may further limit the frequency of generations, which may directly affect population density and even limit its rate of spread [37,38,39]. Therefore, vanillic acid can be selected as a preferred substance for plant-based pesticides and be included in the integrated management system for *S. frugiperda*.

However, compared with vanillic acid, the negative impact on the growth and development of *S. frugiperda* treated with sinapinc acid is less persistent. Although sinapic acid treatment (S_2_, S_3_) can limit weight gain of larvae by affecting their food utilization, the S_3_ treatment did not have a significant effect on the development of larvae and pupae. The S_2_ treatment did significantly prolong the development of the sixth instar larvae and pupae. This indicates that the early application of sinapic acid at the second instar can delay the tolerance period of *S. frugiperda*. Even so, this influence also did not persist into the adult stage. Instead, it might have led to the self-repair and compensation of *S. frugiperda*, such as the phenomenon of an extended longevity for the adult. Phenolic substances such as sinapic acid are widely present in host plants of *S. frugiperda* such as ryegrass [26]. Therefore, in the chemical defense process of such plants, the synthesis rate of phenolic substances is likely to become a potential factor affecting the effectiveness of chemical defense.

The results of principal component analysis showed that the addition of vanillic acid or sinapic acid at both the second and third instar stages of *S. frugiperda* larvae resulted in a high degree of separation from the control. The separation of PC1 between the vanillic acid (V_2_, V_3_) treatment group and the control group is mainly related to two variables: the food intake and body weight of the larvae. In the separation between the sinapic acid (S_2_, S_3_) treatment group and the control group, the variables that contributed significantly were the MFO activity of larvae, food conversion rate, and utilization rate. This indicates that the direct toxic effects of vanillic acid and sinapic acid on the *S. frugiperda* are at the same stage, mainly concentrated in the larval stage. However, there are certain differences in the toxic characteristics caused by the two phenolic compounds, which are likely the key reason for the different persistence of toxic damage to the *S. frugiperda*.

It is worth noting that, compared to laboratory environments, field conditions are more complex. Therefore, in order to accelerate the application of plant-based additives in commercial-scale cultivation, it is necessary to further improve their effectiveness and sustainability. The chemical defense of plants against pest invasion often involves the joint participation of multiple phenolic substances [40]. There may be a synergistic effect between these substances. This research provides ideas for the next steps, improving the effectiveness of plant-based additives. In addition, this problem could possibly be solved by means of microcapsule technology. Appropriate microcapsule materials can enhance the stability and release speed of the core substance, hereby effectively enhancing the sustained effectiveness of control [41]. This may further improve the safety of plant-based additives, but this still needs to be verified through research.

## 5. Conclusions

**Figure 13:**
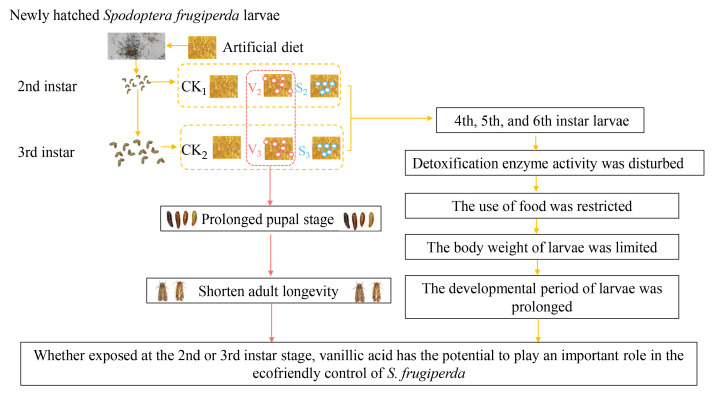
Evaluation of the toxic effects of exposure to phenolic compounds on *S. frugiperda*.

The effects of exposure to vanillic acid on the larvae, pupae, and adult longevity of *S. frugiperda* in the second or third instar stage were relatively similar. Both stimulated the 4th-6th larvae to activate detoxification enzyme defense mechanisms, and had adverse effects on their food utilization rate, food intake, body weight, and developmental duration. *S. frugiperda* has a relatively strong tolerance to sinapic acid, and exposure to sinapic acid at the second or third instar stage did not have adverse effects on the adult longevity. Therefore, compared with sinapic acid, whether at the second or third instar stage of *S. frugiperda*, exposure to vanillic acid has the potential to play an important role in its ecofriendly control (Figure 13). This study provides an effective solution for the comprehensive control strategy of *S. frugiperda*. However, the wild environment is comparatively complex, so using either synergistic effect, phenolic substances or microcapsule technology, to improve the effectiveness of plant-based additives has become the focus of further research.

## Figures and Tables

**Table 1:** Schematic for treatment and sampling stages.

Group Code	Plant-Based Additives	Larvae Treated	Sampling Stages	
			Enzyme Activity, Food Intake, Food Utilization, Body Weight	Developmental Duration
CK_1_	-	-	newly molted 4th, 5th, and 6th instar larvae	4th, 5th, 6th instar larvae, pupae, and adults
V_2_	Vanillic acid	2nd to 6th instar		
V_3_	Vanillic acid	3rd to 6th instar		
CK_2_	-	-		
S_2_	sinapic acid	2nd to 6th instar		
S_3_	sinapic acid	3rd to 6th instar		

## Data Availability

The datasets generated during and/or analyzed during the current study are available from the corresponding author on reasonable request.
